# Quantifying the Suitability of Biosignals Acquired During Surgery for Multimodal Analysis

**DOI:** 10.1109/OJEMB.2024.3379733

**Published:** 2024-03-20

**Authors:** Ennio Idrobo-Ávila, Gergő Bognár, Dagmar Krefting, Thomas Penzel, Péter Kovács, Nicolai Spicher

**Affiliations:** Department of Medical InformaticsUniversity Medical Center Göttingen, Georg-August-Universität 27177 37075 Göttingen Germany; Department of Numerical Analysis, Faculty of InformaticsEötvös Loránd University 272352 1117 Budapest Hungary; Interdisciplinary Center of Sleep MedicineCharité - Universitätsmedizin Berlin 14903 10117 Berlin Germany

**Keywords:** Signal quality, physiological signals, VitalDB dataset, SIESTA dataset, multimodal analysis

## Abstract

*Goal:* Recently, large datasets of biosignals acquired during surgery became available. As they offer multiple physiological signals measured in parallel, multimodal analysis – which involves their joint analysis – can be conducted and could provide deeper insights than unimodal analysis based on a single signal. However, it is unclear what percentage of intraoperatively acquired data is suitable for multimodal analysis. Due to the large amount of data, manual inspection and labelling into suitable and unsuitable segments are not feasible. Nevertheless, multimodal analysis is performed successfully in sleep studies since many years as their signals have proven suitable. Hence, this study evaluates the suitability to perform multimodal analysis on a surgery dataset (VitalDB) using a multi-center sleep dataset (SIESTA) as reference. *Methods:* We applied widely known algorithms entitled “signal quality indicators” to the common biosignals in both datasets, namely electrocardiography, electroencephalography, and respiratory signals split in segments of 10 s duration. As there are no multimodal methods available, we used only unimodal signal quality indicators. In case, all three signals were determined as being adequate by the indicators, we assumed that the whole signal segment was suitable for multimodal analysis. *Results:* 82% of SIESTA and 72% of VitalDB are suitable for multimodal analysis. Unsuitable signal segments exhibit constant or physiologically unreasonable values. Histogram examination indicated similar signal quality distributions between the datasets, albeit with potential statistical biases due to different measurement setups. *Conclusions:* The majority of data within VitalDB is suitable for multimodal analysis.

## I. Introduction

Biosignals such as electrocardiography (ECG) or electroencephalography (EEG) reflect physiological functions and are used for deriving clinically relevant parameters in various settings. Several decades ago, biosignal monitoring shifted from measuring single signals to monitoring multiple signals in parallel. In case these signals stem from different measurement modalities, these measurements are entitled as being “multimodal”. Multimodal biosignal analysis finds wide-ranging applications such as cuff-less blood pressure estimation [1], [2], cardiorespiratory coupling analysis [3], stress monitoring [4], sleep assessment [5], [6], network analysis of organ interaction [7], and monitoring in intensive care units [8].

The publication of the MIT-BIH Arrhythmia Database in the 1980 s pioneered biosignal processing research [9]. Until then, the assessment of algorithms was not reproducible as evaluation was performed on proprietary data by medical device vendors. Other datasets of unimodal biosignals, such as the European ST-T Database [10], and eventually multimodal measurements, such as the SIESTA dataset [11], followed. Today, the PhysioBank offered by PhysioNet is an extensive dataset of biosignals from healthy subjects as well as patients suffering from diverse diseases [12]. Recently, multiple large-scale datasets offering multimodal biosignals were made publicly available, e.g. including surgery patients [13], hospital patients [14], and patients undergoing sleep studies [15]. The data has been used for different tasks, e.g. blood pressure estimation [16], prediction of massive blood transfusion [17], and decision support in sepsis treatment [18]. However, despite the fact these fields are different in principle, they face the common issue related to the so-called “quality” or “suitability” of the data. Both concepts describe the relationship between the underlying physiological signal and unwanted noise components. In the remainder of this work, we use both concepts synonymously; a signal of high quality (low noise) is suitable for analysis and a signal of low quality (high noise) is unsuitable for analysis.

In contrast to early datasets that were carefully labelled and visually inspected [9], [10], [11], in current datasets, manual labelling of the data in suitable and unsuitable segments is often difficult or even impossible to conduct due to the sheer amount of data. This trend is further driven by the escalating demand for data by deep learning techniques. This underscores the critical significance of conducting suitability studies in these large-scale datasets. Outside the research field of biosignal processing, the signal-to-noise ratio (SNR) is often used to quantify signal quality. However, this approach requires a clear definition of what parts of a signal are associated with the physiological function of interest and what are associated with noise. This concept cannot be mapped directly to biosignals as the characteristics of signal and noise are often similar, i.e. they fall within the same frequency ranges. For example in ECG, motion artifacts can distort the ST segment, power-line inference can distort the P-wave, and the frequency of electromyography (EMG) noise considerably overlaps with the main ECG signal [19].

Therefore, in biosignal analysis, signal quality indicators (SQIs) have been proposed to indicate the quality of a signal; e.g. based on frequency content in different bands and out-of-range events [20], kurtosis and the proportion of the spectral distribution [21], morphological, statistical and spectral characteristics [22], relative power in the QRS complex, skewness, percentage of the signal with a flat line appearance [23], constant-values and QRS detection [24]. In the majority of cases, the proposed metrics are specific to a single class of biosignal, e.g. for pulse oximetry [25], EMG [26], or ECG [27]. In addition, a wide variety of SQIs were introduced for data acquired with wearable devices and targeting motion artifacts as they are likely to contain them [22], [25], [27].

In surgical practice, signal quality is highly relevant as noise produces serious issues, such as increased false alarm rates causing decreased quality of care due to alarm fatigue [22]. Typical sources of noise are power-line interference, motion artifacts due to surgical preparation and patient movement, improper sensor contact, and leaks in ventilator units. An in-depth review can be found in [28]. Recently, large datasets of biosignals acquired intraoperatively became available, that might offer novel insights into physiological processes [13], [29], [30] with VitalDB being the largest and most diverse collection of biosignals available at the moment [13]. However, it is unclear what percentage of this data is suitable for multimodal analysis as multiple thousands of surgeries cannot be manually labelled.

Hence, in this work, we apply existing SQIs to quantify the suitability of VitalDB [13] for multimodal analysis. As there are no multimodal SQIs available, we use unimodal signal quality indicators only. In case, all signals within a time segment are determined as being of high quality by their respective SQIs, we assume that the whole segment is suitable for multimodal analysis. In the same way, SIESTA is analyzed; this was acquired in sleep laboratories [31] as a normative polysomnography dataset and has been the subject of multiple multimodal analyses [7], [32] before, demonstrating its suitability. Therefore, we take SIESTA as a reference and are interested in what level of suitability VitalDB reaches.

The datasets are similar with respect to subject conditions as they are i) not awake the majority of the time, ii) lying down, and iii) not performing large movements. Due to the different environments, the datasets contain similar but also different modalities. We apply the SQIs to the common biosignals available in both datasets, i.e. ECG and EEG, while respiration signals (Resp) are measured using different modalities.

## II. Materials and Methods

VitalDB is a free and comprehensive dataset that contains intraoperative biosignals and clinical information on 6388 surgical patients [13]. This dataset includes waveform signals, numeric values, and surgery-related clinical information. Data was collected in the years 2016-2017 within the Seoul National University Hospital from patients undergoing routine or emergency surgery for surgery other than cardiac (urologic, gynecologic, thoracic, and general). The corresponding article states that it contains noise from the following categories: data loss due to sensor detachment, abnormal values, noise during electrocautery and power-line inference [13]. The authors of this work are not aware of any systematic external signal quality analysis of VitalDB. It is freely available under a CC BY-NC-SA 4.0 license.

The SIESTA dataset includes polysomnography recordings of 197 healthy individuals and 97 individuals with high-prevalence sleep disorders, e.g. sleep apnea, that were recorded in eight European sleep centers [31]. In most cases, two consecutive nights were recorded, resulting in a total of 669 polysomnography records. Sampling rates vary between the different sleep centers due to different equipment being used [11]. An unimodal analysis of signal quality has been conducted for the SIESTA study [11] based on histogram and entropy analysis, revealing issues such as clipping of signal amplitudes, drifts, zero or out-of-range values.

### A. Collected Datasets

Both datasets consist of different methods for biosignal acquisition, depicted in Table I. We included the common signals, namely ECG and EEG signals. While there is only a single-lead ECG in SIESTA available, two leads are available in VitalDB. After inspecting the data, we chose lead II as the other lead was often not in use. Regarding EEG, in VitalDB a bifrontal montage of a device for depth of anesthesia monitoring is used. In order to make that as comparable as possible, we selected Fp1-M2 and C3-M2 EEG channels for SIESTA. To account for respiration, oro-nasal airflow signals from SIESTA and the capnography signals from VitalDB were included. The latter is a non-invasive technique used to monitor the level of carbon dioxide in exhaled breath and to measure the patient's respiratory status [33], [34]. The technique involves the use of sensors detecting concentration of CO$_{2}$ in the airway [33], [34]. In contrast, airflow sensing is another technique used to monitor respiratory status which involves the use of sensors that detect the flow of air in and out of the lungs. There are several types of airflow sensors [35] and within SIESTA airflow is measured with a sensor at the nose. The inclusion of both measurements stems from the potential to present a robust method for evaluating two distinct signals associated with the same physiological process — respiration, in this instance.

**TABLE I:** Physiological Signals of VitalDB and SIESTA Datasets

VitalDB	SIESTA
ECG - two channels	ECG
EEG - two channels	EEG - eight channels
Airway pressure - capnography	Respiratory airflow
PPG	Respiratory effort
Blood pressure	Electromyogram
Respiration	Electrooculogram - two channels
Flow wave	

#### 1) Data Acquisition

VitalDB signals were downloaded using the provided Python library available via the *Python Package Index (PyPI)* [36]. The SIESTA data is available in European Data Format (.edf) files which were read using the *edfrd library* [37] which is also available via PyPi.

#### 2) Exclusion and Inclusion Criteria

The whole datasets were included in both cases, SIESTA and VitalDB. For the SIESTA dataset, all 391 subjects were included (669 records, 9388.01 hours of recordings), while for VitalDB, all 6388 subjects were included (40247.48 hours of recordings). To avoid adding a bias to the data, we did not adjust the size of datasets by balancing their number of records or patients and instead give all results in percentage values.

#### 3) Data Preprocessing

Signals in VitalDB have different sampling rates with respect to the type of signal. ECG, EEG, and capnography signals were recorded with a sampling rate of 500, 128, and $62.5 \,\text{Hz}$, respectively. In contrast, SIESTA has different sampling rates not only across the different types of signals but also within the same type of signals. ECG signals are available with sampling rates of 400, 256, 200, and 100 Hz while EEG signals have 256, 200, and 100 Hz as sampling rates; airflow data was acquired using 256, 200, 100, 25, 20, and 16 Hz as sampling rates.

We did not apply any resampling to the signals as this would bias the results of some SQIs. In addition, we did not apply any preprocessing in terms of signal filtering, baseline removal, etc. The rationale here was to provide a bottom base-line with respect to signal quality. Of course, researchers can use signal filtering when working with VitalDB due to the overlap of signal and noise components, nonetheless this often degrades the signal as well [38] and may even distort diagnostic information [39]. Hence, the only preprocessing step was to split each record into segments of ten seconds with an overlap of five seconds.

### B. Signal Quality Indicators

Some methodologies employed in our previous applications were adapted for the analysis presented in the current study [40]. We employed two types of generally applicable SQIs: rule-based and statistical indicators. The first checks signals for completeness, constant data not containing any informative value, and if signal amplitudes are in the expected physical range. These rules cover the types of noise that were analyzed in earlier work on the SIESTA study [11]. The second type of SQIs includes the computation of statistical measures such as skewness, kurtosis, entropy, zero-crossing rate, and the standard deviation (std) of variations in signal envelopes, both higher and lower. Additionally, we used a third type of SQI from literature based on direct physiological assumptions which exist for ECG signals only.

#### 1) Rule-Based SQIs

Each rule-based SQI results in a boolean output for each ten-second segment of each signal, reflecting whether the quality is high, or not.

*Constant data:* Biosignals are almost always changing due to the dynamic nature of physiological processes within the human body and hence are rarely constant for long durations. If a sustained period of constant value is observed, it is almost always the result of sensor contact loss or clipping artifacts. Thereby, it was analyzed if a segment exhibited constant values over periods of 500 ms or longer. More precisely, a segment is considered to have constant data, if the condition

\begin{equation*}
x_{i} = x_{i+1} = \ldots = x_{i+w_{\text{length}}-1} \tag{1}
\end{equation*}

holds for any sample index $i = 0,1,\ldots,N-w_{\text{length}}$, where $N$ represents the number of samples in a signal $x$, and $w_{\text{length}}$ refers to the number of samples in time period of 500 ms. This approach was applied similarly as described in [41].

*Out-of-range data:* Out-of-range values might occur mainly due to measurement hardware-related issues or problems during data conversion. In each segment, their respective maximum and minimum values were compared to values categorized as normal in clinical environments which act as thresholds for this score. The considered upper and lower thresholds were retrieved from literature and defined as intervals: $[-110,110] \mu \text{V}$ for EEG signals [42], $[-3.5,3.5] \text{mV}$ for ECG signals [43], and $[0,50] \text{mmHg}$ for the capnography signal [44]. As the respiration signal in SIESTA was dimensionless, establishing an acceptable range was not possible.

#### 2) Statistical SQIs

Each statistical SQI results in a numerical output for each segment of each signal.

*Standard deviation of the upper and lower envelope:* Analysis of signal envelopes allows for detecting noisy peaks in a signal and has already been used for assessing the quality of pulse oximetry signals [45]. At first, signal amplitudes were scaled to $[-1,1]$ to normalize across the different types of signals. Subsequently, the ten-second segments were split into smaller segments (ECG, EEG: two seconds, Resp: five seconds), maximum and minimum values were extracted, and their std was computed.

\begin{equation*}
\sigma (x) = \sqrt{\frac{\sum _{i=1}^{N}(x_{i}-\bar{x})^{2}}{N-1}} \tag{2}
\end{equation*}

*Skewness:* The skewness of a signal is associated with how much the distribution of amplitudes deviates from a normal distribution and is thereby a measure of asymmetry. Skewness was used as a metric of quality in different types of signals such as pulse oximetry [46], ECG [47], and EEG [48]. Skewness is defined by

\begin{equation*}
\,\mathit{Skewness}(x) = \frac{1}{N}\sum _{i=1}^{N}\left[ \frac{\left(x_{i}-\bar{x} \right) }{\sigma } \right]^{3} \tag{3}
\end{equation*}

where $\bar{x}$ represents the mean value of the signal, $\sigma$ represents the std, and $x_{i}$ is the amplitude of the $i$-th sample.

*Kurtosis:* Kurtosis is an indicator of whether a given signal contains extreme values or not. It identifies how much the tails of the signal's distribution differ from the tails of a normal distribution. In the same manner as skewness, kurtosis has been implemented as an element for assessing the quality of signals such as pulse oximetry [49], ECG [47], and EEG [48]. Kurtosis is defined by

\begin{equation*}
\,\mathit{Kurtosis}(x) = \frac{1}{N}\sum _{i=1}^{N}\left[ \frac{\left(x_{i}-\bar{x} \right) }{\sigma } \right]^{4} \tag{4}
\end{equation*}

*Entropy:* Entropy is a measure of unpredictability or randomness since it quantifies how much the probability density function of a signal varies from a uniform distribution [48]. It has been used successfully for measuring signal quality of pulse oximetry [49], ECG [50], and EEG [48] signals. Here we used the spectral entropy of the form

\begin{equation*}
\mathit{Entropy}(x) = -\frac{1}{\log _{2}\,M}\sum _{i=1}^{M} p(x) _{i} \log _{2} \left(p(x) _{i} \right) \tag{5}
\end{equation*}

where $M$ is the number of frequency bins, and $p$ is the normalized power spectral density of signal $x$. The scaling factor $\log _{2}\,M$ refers to the maximal spectral entropy of white noise. In this sense, segments with a high entropy are expected to mostly consist of noise and segments with a very low entropy are probably missing physiological waveforms.

*Zero-crossing rate:* A zero-crossing occurs in a signal when a signal changes from positive to negative sign or vice versa. Thereby, the zero-crossing rate was included as a simple method for detecting the presence of noise in the signals as high zero-crossing rates are associated with the presence of noise, e.g. high-frequency sawtooth signals. This approach was already used in other works for ECG [27], photoplethysmographic (PPG) [25], and EEG signals [51]. The zero-crossing rate is defined by

\begin{equation*}
\begin{split}& \mathit{Zero-crossing\, rate}(x) = \frac{1}{\left(N-1 \right) } \sum _{i=1}^{N} \mathbb {I} \left(x_{i}x_{i-1} < 0 \right), \\
& \text{ where } \mathbb {I} = 1 \text{ if } x_{i}x_{i-1} < 0, \text{ otherwise } \mathbb {I} = 0. \end{split} \tag{6}
\end{equation*}

This approach assumes that biological signal content is commonly in a limited low band of frequencies (i.e. 0 to 30 Hz for EEG [52], and 0.05 to 100 Hz for ECG [53], with dominant components in the range $< 30$ Hz [54]), while noise commonly is present in the whole band of frequencies, exhibiting elevated levels of both frequency and energy [53].

#### 3) Physiological SQIs

The SQIs introduced so far are based on quantitative properties of a certain signal but not on physiological meanings. Only a few approaches specific to ECGs have been developed to address this limitation. Therefore, we extend our ECG signal quality analysis with a set of physiologically relevant conditions. According to the survey by Satija et al. [27], a broad spectrum of physiological SQIs exists, which integrates fiducial features with heuristic rules. These methods extract a variety of morphological and interval features, such as the duration and amplitude of the P- and T-wave, QRS complex, PR and ST-segments, which are then combined with heuristic rules using predefined physiologically relevant decision thresholds. In our study, we selected heuristic rules that operate based on the location of the R-peak, considering it to be one of the most robust features that can be automatically detected among those mentioned previously as we have no insights into ECG signal quality. We applied the three conditions introduced by Orphanidou et al. [55], specifically designed for scenarios with many motion artifacts as these are also frequently observed in surgery data [28].

Condition 1 – Feasibility of the estimated heart rate (HR) - It must be in a physiologically probable range that is between 40 and 180 beats per minute for the adult population.

Condition 2 – Maximal distance between two QRS complexes: the longest time interval between two consecutive R-peaks cannot exceed 3 s, thus no more than one beat can be missed.

Condition 3 – Relative change of the HR: The ratio of the maximum to the minimum beat-to-beat interval cannot exceed 2.2. This rule ensures that the HR cannot change by more than 10%, provided that no more than a single beat has been missed.

In the present analysis, we applied conditions 1–3 to each ten-second segment of the ECG recordings [56]. First, we applied three different QRS detectors, namely ProMAC developed by NeuroKit2 [57], the algorithm proposed by [58], and the QRS detector from the ECG-kit Matlab toolbox [59]. Second, each condition was checked based on the detected QRS complexes. A single condition was evaluated three times, i.e. once for every QRS detector. For each rule, the final decision was made based on majority voting. If any of the feasibility conditions were violated, the corresponding signal segment was considered of bad quality.

### C. Signal Analysis Methodology

We aimed to determine the suitability of the datasets for unimodal and multimodal analysis, and to identify the similarities and differences between the suitability of the two datasets. Multiple aspects of analysis were performed based on the statistical behaviour of the SQIs.

#### 1) Unimodal and Multimodal Analysis

Here we investigated the mean statistical behaviour of the SQIs, and determined the amount of suitable for analysis considering single biosignals (unimodal analysis), and considering multiple synchronous signals (multimodal analysis). On one hand, for unimodal analysis, only one signal at once is considered for assessing its suitability. For this analysis, all indicators were included, i.e. rule-based, statistical, and physiological SQIs; the physiological SQIs were applied exclusively to ECG signals. On the other hand, for multimodal analysis, two or more signals at once are included to evaluate their suitability. In this approach, only rule-based SQIs were employed for the analysis. These methods were selected to allow a more direct evaluation including different amounts of signals. In this way, multimodal analysis was carried out considering all of the possible combinations for analysis among two, three, and four signals in each dataset.

#### 2) Histogram Analysis

Besides the unimodal and multimodal suitability analysis, we conducted a cross-dataset consistency evaluation. We investigated the similarities and differences between the two datasets according to a histogram-based analysis and comparison of the SQIs. Both qualitative and quantitative analyses were performed, i.e. we visually evaluated the distributional properties of the histograms, and then we estimated the effects between the histograms of the two datasets. We noted that statistical hypothesis testing is also a common approach to comparing two populations, but it is impractical here due to the large sample size. The standard statistical tests (like *t*-tests, *F*-tests, Kolmogorov–Smirnov test, etc.) may detect even small differences as significant in large samples, referred as the *p*-value problem [60]. Due to this, we focused on the practical differences instead of statistical significance, and we estimated the effect sizes, i.e. we measured the strength of the relationship between the histogram pairs by quantifying the correlation coefficients.

In general, the selected physiological signals are expected to have similar characteristics for both datasets, so we also anticipated similarities between the SQIs as well. However, the datasets have certain differences in terms of technical setup (e.g. devices, sensors, settings, and calibrations) and medical conditions of patients, which may cause bias or other statistical differences between the SQIs. In particular, we expected entropy differences between the two datasets, due that unlike the other statistical SQIs, the entropy depends on the sampling rate of the signal. Considering the sampling rate differences between the datasets, it is reasonable to match the histograms before further comparison. For matched histogram analysis, the means and the variances of the entropy SQIs from the SIESTA dataset are matched to those from VitalDB.

## III. Results

Results are presented in this section according to the type of analysis: unimodal, multimodal, and histogram analysis.

### A. Unimodal Analysis

#### 1) Rule-Based SQIs

Table II displays the results of the rule-based SQIs being applied to single biosignals of both datasets; values are presented as the mean of percentage values. It can be noted that regarding constant signal amplitudes, SIESTA involves a mean of 0.30% compared to 8.37% in VitalDB. In addition, 14.25% of SIESTA signal amplitudes are out of the predefined range, in contrast to 15.63% of VitalDB. VitalDB shows a high number of constant data, however, both datasets present a similar proportion of out-of-range data.

**TABLE II:** Results for Rule-Based SQIs: Percentage of Constant Values, and Out-of-Range Data for Both Datasets

Signal	Constant data (%)				Out-of-range data (%)			
	VitalDB		SIESTA		VitalDB		SIESTA	
EEG 1	10.37		0.44		24.85		24.30	
EEG 2	10.37		0.27		17.79		15.14	
ECG	1.88		0.15		3.68		3.32	
Resp	10.85		0.35		16.21		—-	
Mean	8.37		0.30		15.63		14.25	

#### 2) Statistical SQIs

The mean computed by the statistical SQIs introduced in Section II-B were computed for VitalDB and SIESTA datasets and shown in Table III. This analysis shows a difference between the outcomes from both datasets in the features of skewness and kurtosis. However, the zero-crossing rate provides a similar behaviour across all signals for both datasets. In the same manner, the upper and the lower envelopes present similar values for EEG1, EEG2, and ECG in VitalDB as well as SIESTA.

**TABLE III:** Results for Statistical SQIs: For Each Record, the Arithmetic Mean Over All segments is Computed

Signal	Skewness		Kurtosis	
	VitalDB	SIESTA	VitalDB	SIESTA
EEG 1	0.02$\pm$1.88	0.15$\pm$0.75	8.59$\pm$32.09	7.50$\pm$5.00
EEG 2	0.05$\pm$1.16	0.05$\pm$0.54	8.54$\pm$35.99	7.10$\pm$5.17
ECG	2.17$\pm$1.78	1.20$\pm$2.77	13.42$\pm$30.27	22.92$\pm$12.50
Resp	-0.26$\pm$0.59	-0.02$\pm$0.61	1.64$\pm$5.87	5.44$\pm$4.10
				
Signal	Entropy		Zero-crossing rate	
	VitalDB	SIESTA	VitalDB	SIESTA
EEG 1	0.54$\pm$0.15	0.52$\pm$0.13	0.11$\pm$0.10	0.19$\pm$0.13
EEG 2	0.54$\pm$0.16	0.60$\pm$0.12	0.11$\pm$0.10	0.20$\pm$0.13
ECG	0.50$\pm$0.13	0.69$\pm$0.09	0.03$\pm$0.07	0.04$\pm$0.04
Resp	0.18$\pm$0.10	0.28$\pm$0.15	0.01$\pm$0.01	0.09$\pm$0.26
Signal	Upper envelope		Lower envelope	
	VitalDB	SIESTA	VitalDB	SIESTA
EEG 1	0.20$\pm$0.12	0.23$\pm$0.11	0.21$\pm$0.10	0.21$\pm$0.10
EEG 2	0.21$\pm$0.10	0.20$\pm$0.09	0.21$\pm$0.10	0.19$\pm$0.09
ECG	0.08$\pm$0.09	0.05$\pm$0.05	0.08$\pm$0.11	0.04$\pm$0.05
Resp	0.03$\pm$0.12	0.10$\pm$0.12	0.01$\pm$0.07	0.11$\pm$0.14

#### 3) Physiological SQIs

The physiological SQIs were evaluated for ECG signals in both datasets, and the data segments were labelled as good or bad quality. Table IV presents the statistics of the physiological indices. This analysis shows that the general signal quality of the two datasets is in a similar range, although the SIESTA dataset has a higher percentage of good-quality data.

**TABLE IV:** Results for Physiological SQIs: Percentage of Good and Bad Quality Segments for Both Datasets

Signal	Good quality (%)			
	VitalDB		SIESTA	
ECG	78.22		87.77	

### B. Multimodal Analysis

Table V displays the results of multimodal analysis considering the combinations of two biosignals in a given segment. We assumed a combination as suitable for analysis if both signals satisfy the rule-based SQIs, i.e. they do not show constant, or out-of-range data in the segment. For VitalDB the percentage of data which might be used for multimodal analysis is 72% and for SIESTA 82%. The percentage of suitable data that involves respiratory signals is higher in SIESTA, and the percentage which does not include them is similar in both datasets.

**TABLE V:** Percentage of Data With No Constant Values and Amplitudes Within Normal Ranges*

Signal	VitalDB (%)			SIESTA (%)		
	EEG 2	ECG	Resp	EEG 2	ECG	Resp
EEG 1	74.47	72.69	57.15	73.26	75.31	75.49
EEG 2	—	79.45	62.50	—	84.43	84.60
ECG	—	—	71.43	—	—	96.22
Mean $\pm$ std	72.11$\pm$5.52			81.55$\pm$7.93		

Table VI shows results for combinations of three and all four signals which might be used for multimodal analysis. It can be noted that the amount of data which might be used for multimodal analysis with three signals is larger than 60%. For VitalDB the percentage is 61% and for SIESTA 76%. The percentage of the data that involves respiratory signals is higher in SIESTA, and the percentage which does not include them is similar in both datasets. It is possible to observe that the amount of data which might be used for multimodal analysis with four signals is bigger than 55%; namely 55% for VitalDB and 73% for SIESTA.

**TABLE VI:** Percentage of Data With No Constant Values and Amplitudes Within Normal Ranges*

Signal	VitalDB (%)			SIESTA (%)		
EEG1, EEG2, ECG	72.08			72.91		
EEG1, EEG2, Resp	56.69			73.08		
EEG1, ECG, Resp	55.45			75.13		
EEG2, ECG, Resp	60.57			84.19		
Mean $\pm$ std	61.20$\pm$6.56			76.33$\pm$4.62		
Signal	VitalDB (%)			SIESTA (%)		
EEG1, EEG2, ECG, Resp	55.01			72.74		

### C. Histogram Analysis

**Fig. 1.**
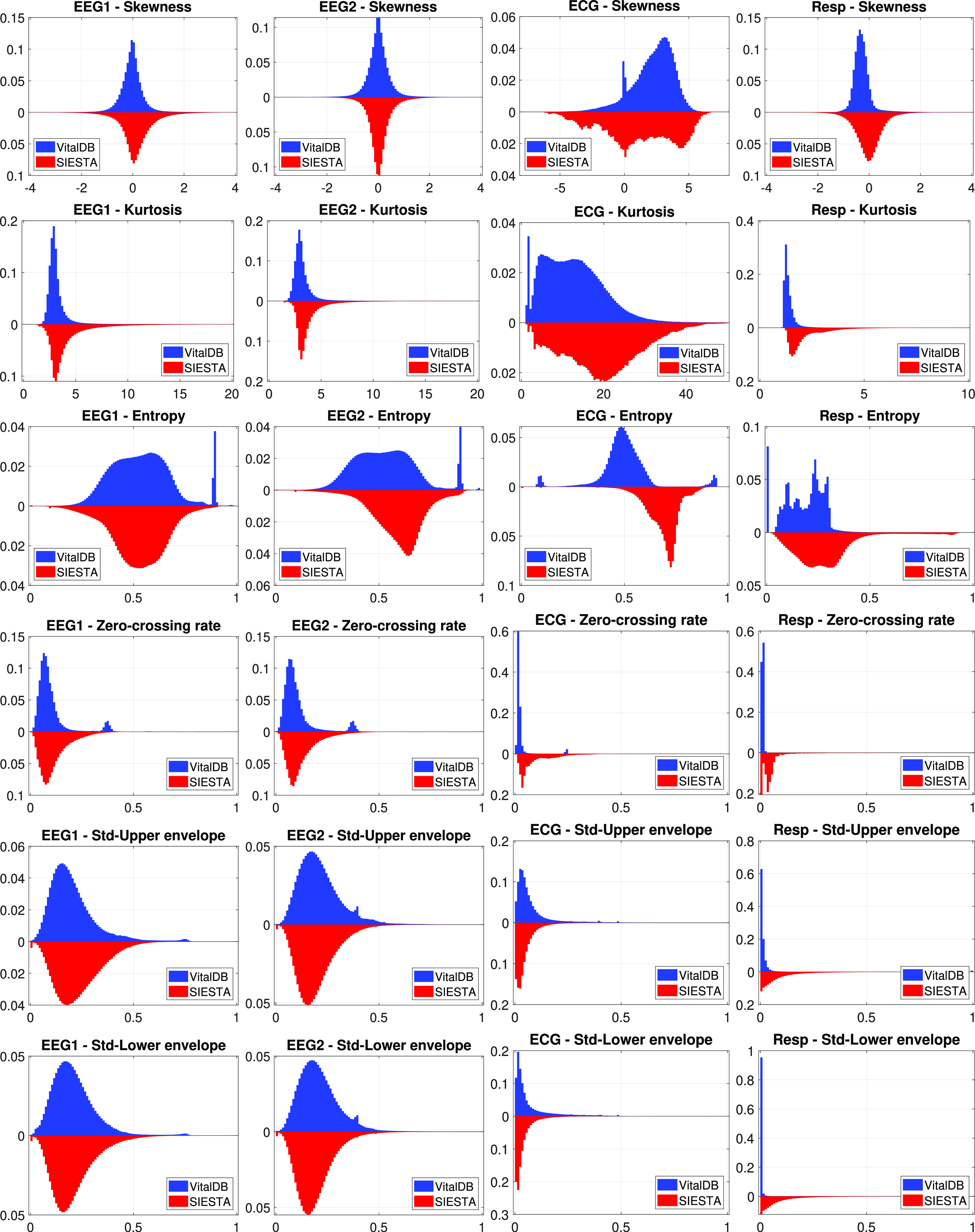
Back-to-back histograms of the SQIs, normalized with the sample sizes. Horizontal and vertical axes represent SQI values and normalized frequencies, respectively.

We performed a histogram-based analysis of the statistical SQIs. Fig. 1 represents the back-to-back histograms of SQIs, i.e. the vertical combinations of the histogram pairs from the two datasets. The histograms were computed for the whole datasets, i.e. the histograms were the aggregates of all segments. Note that kurtosis and skewness are not bounded metrics, while the other SQIs were normalized to interval [0, 1]. Here we chose the display ranges [0, 20] for EEG kurtosis, [0, 50] for ECG kurtosis, [0, 10] for respiratory kurtosis, $[-4,4]$ for EEG skewness, $[-8,8]$ to ECG skewness, and $[-4,4]$ for respiratory skewness, in order to provide better visual interpretability. With few exceptions, to be discussed later, the histogram pairs had similar behaviour in terms of shape and symmetry. However, as it is already depicted in Table III, there were differences between statistical descriptors, like mean and variance, that may cause bias between these histogram pairs (see e.g. EEG 2 and ECG entropy on Fig. 1). In general, we can conclude that the SQIs of these signals usually followed similar statistical distributions for both datasets, the differences come from the technical differences of the two datasets.

The quantified correlation coefficients of the histogram pairs are presented in Table VII. Here, we investigated the quantitative effect sizes between the histogram pairs by estimating the correlation between the histogram curves. Correlation coefficients were evaluated on histograms quantized with 1000 bins. The usually high correlation numbers (close to 1) also indicate considerable similarity between the datasets.

**TABLE VII:** Correlation Coefficients of the SQI Histogram Pairs

Signal	Skewness	Kurtosis	Entropy	Zero-crossing	Upper	Lower
				rate	envelope	envelope
EEG 1	0.94	0.86	0.89	0.88	0.94	0.99
EEG 2	1.00	0.88	0.68	0.87	0.99	0.98
ECG	0.65	0.43	0.16	0.31	0.92	0.97
Resp	0.72	0.37	0.38	0.14	0.64	0.50

**Fig. 2.**
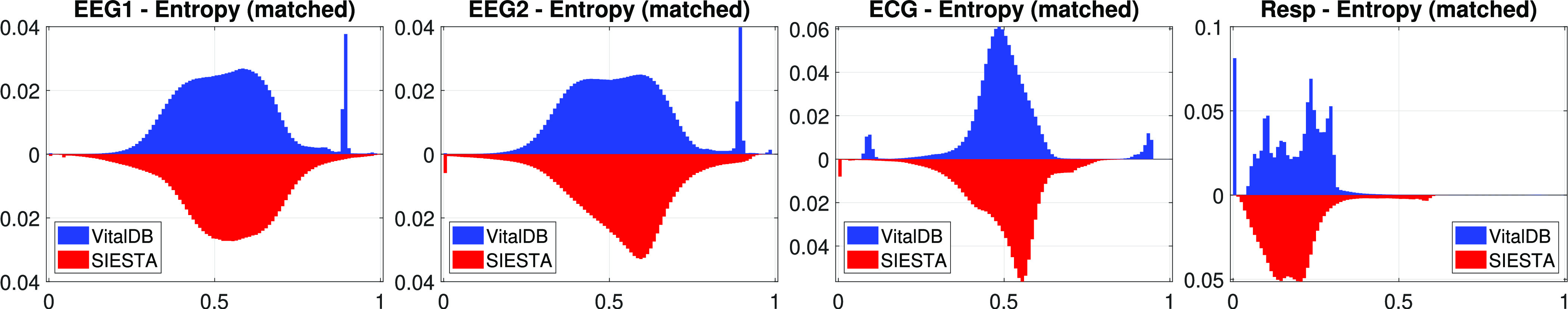
Matched back-to-back histograms of the entropy indices for all signals. Horizontal and vertical axes represent SQI values and normalized frequencies, respectively.

Let us now focus on the exceptional cases, where the histogram pairs have visual dissimilarities, and/or there is only a low level of correlation between them. The most notable cases are the SQIs of the Resp signals and the entropy indices of the signals. We note that the respiratory signals were derived from different modalities (capnography vs. airflow, see Section II-A). The differences between the device sensors and the monitoring techniques might explain the dissimilarities of the SQIs. Regarding the entropy histograms, similar shapes but different means and variances may be observed. Considering that entropy might depend on the sampling rate, we matched the histogram before further comparison, as of Section II-C. Fig. 2 demonstrates the matched histogram pairs. The correlation coefficients then became 0.88, 0.84, 0.82, and 0.39 for EEG 1, EEG 2, ECG, and Resp, respectively, which indicates correspondence between the entropies of the EEG and ECG signals.

Another remarkable difference between the datasets is that there are more SQI outliers in VitalDB than in SIESTA (see e.g. the secondary and ternary peaks of the entropy histograms in Fig. 1). We investigated this behaviour further, and we found a connection between the entropy outliers of the ECG signal and the physiological SQIs with the entropy outliers almost always indicating low signal quality.

**Fig. 3.**
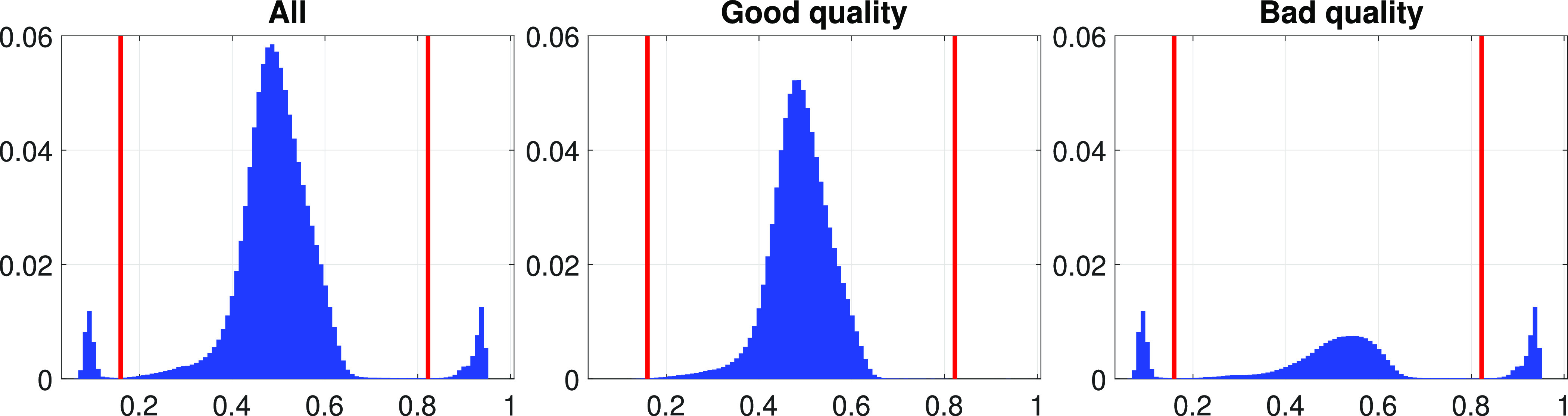
ECG entropy histograms corresponding to the physiological SQIs. The red vertical lines are the automatically computed outlier thresholds. Horizontal and vertical axes represent SQI values and normalized frequencies, respectively.

Fig. 3 shows the ECG entropy histograms computed for good and bad quality segments, based on the physiological SQIs. We can observe that both the lower and upper outliers correspond to bad-quality signals. We also estimated automatic thresholds to detect the outliers of the entropy histogram by finding the minimum points between the three peaks (see Fig. 3, right panel), and we evaluated the performance of these thresholds against the physiological SQIs. Although this approach detects only 34.70% of the bad-quality segments, the precision is 99.43%, i.e. almost every entropy outlier segment is of bad quality.

## IV. Discussion

In this section, results are discussed in the same order as the previous section: unimodal analysis, multimodal analysis, and histogram analysis. Subsequently, limitations, scope and future work are stated.

### A. Unimodal Analysis

From the unimodal point of view, even though SIESTA presents 0.30% of constant values, only 86% of its data fit the predefined range values (14% is out-of-range). In contrast, VitalDB has more constant data (8%), but its values fit similarly to the predefined amplitude (16% is out-of-range) (Table II).

The statistical SQIs analysis (Table III) showed on one hand that skewness and kurtosis are different between VitalDB and SIESTA datasets. This behaviour might be produced because of the differences in the recording devices and the environment of acquisition. On the other hand, entropy, zero-crossing rate, and the upper envelope and lower envelope SQIs represent robust markers for signal suitability in VitalDB and SIESTA. These markers show a major capability of generalization since their application does not depend on the source of data. Entropy is not affected by the amplitude of data which we could observe in the respiratory signals of both datasets.

In several cases, such as in the zero-crossings of ECG and respiratory (Resp) data, it has been observed that the data points are widely dispersed from the mean value, with the standard deviation often equal to or greater than the mean (Table III). This pattern represents a significant level of variability and dispersion within the data. It is common in medical contexts to encounter datasets that do not conform to the normal distribution. This variability can stem from a variety of factors, including individual differences among patients, the presence of outliers, or the intricate nature of the physiological processes being measured.

### B. Multimodal Analysis

There are several potential applications for performing multimodal analyses on biosignals extracted from both datasets. For example, the concept of cardiorespiratory coupling analyses the coupling between the cardiac and the respiratory systems [61]. The field of network physiology [62] aims for quantifying the coordination and interaction between multiple organ systems, and methods such as time delay stability [7] have already been applied successfully to subgroups of the SIESTA dataset [32]. Our multimodal analysis shows that both datasets have a suitability for multimodal analysis of 72% (VitalDB) and 82% (SIESTA) (Table V) which indicates that the suitability of the surgery dataset is reduced but still the majority is feasible.

It is important to remark that respiratory signals in SIESTA dataset are dimensionless, thus they were not discriminated by the indicator out-of-range data. This might be an explanation for the higher values in multimodal analysis that include these types of signals in SIESTA compared to VitalDB. This is also observed in multimodal analysis for three and four signals (Table VI). The other combinations of two signals, which do not include respiratory signals, present in VitalDB a bigger percentage of data which might be used for multimodal analysis.

The evaluation of the potential for multimodal analysis also reveals a difference between the amount of data in both datasets which might be used. 61% and 76% are associated with VitalDB and SIESTA datasets, respectively for multimodal analysis with three signals, and 55% and 73% for multimodal analysis with four signals (Table VI). In the same way as in multimodal analysis with two signals, the combinations that do not include respiratory signals exhibit in VitalDB a similar percentage of data which might be used for multimodal analysis with three signals.

### C. Histogram Analysis

In order to generalize signal processing methods between datasets, similar or adjustable signal quality is desired. The investigated SQIs were evaluated for cross-dataset consistency using a histogram-based statistical analysis. The analysis revealed dissimilarities between the respiratory signals of the two datasets, and concluded similarity for the EEG and ECG signals, with the note that datasets are biased in terms of mean and variance. This bias can probably be explained by technical differences between the datasets. In summary, we can conclude that the datasets share similar signal quality distributions, but the aforementioned bias should be taken into account during the development of signal processing methods.

The histogram analysis also highlighted the importance of standardization to ensure the internal consistency of the data. This is a natural demand of gradient- and distance-based learning algorithms, since it affects their generalization property [63]. Section III-C showed that some dissimilarities between the datasets due to different measurement setups can be reduced.

An additional revelation is the comparably high number of SQI outliers in VitalDB and their connection to the physiological signal quality. It was already known that the statistical SQIs are capable of differentiating empirical signal quality [25] and that the physiological SQIs also give a reliable depiction of the empirical quality [55]. In this work, we noticed an additional correspondence between entropy outliers and bad physiological quality in ECG signals.

### D. Limitations

We assessed one dataset per clinical environment and assumed that they were representative of the respective environment. As SIESTA was acquired in eight different study centres [31], the results should generalize to a large extent, however, VitalDB was only acquired in a single centre [13] which limits the generalizability of results.

Due to the novelty of VitalDB, we are not able to compare our results to other studies concerning signal suitability. Although there are some works processing this dataset [64], they are i) focussing other biosignals such as photoplethysmography, ii) only processing a subset of VitalDB, and iii) applying preprocessing. Hence, we cannot compare our results to them. Our findings concerning SIESTA align with prior research [11] that analyzed signals individually. This earlier study also addressed issues such as clipping of signal amplitudes, zero values, and out-of-range values, which we detected in our analysis.

Another limitation of our work is that sleep and anaesthesia are two different physiological states. In the realm of the latter, the heightened depression of the central nervous system might introduce notable physiological distinctions when compared to natural sleep. One such disparity manifests in the variance of breathing patterns observed between these two states. This particular dissimilarity might account for certain inconsistencies observed in the analysis, such as discrepancies in respiratory signals during histogram and correlation analysis. These disparities could potentially stem from the collection of data under disparate circumstances. However, paradoxically, this divergence underscores the adaptability and efficacy of the current methodology. It demonstrates the method's ability to be successfully applied even when dealing with data acquired under diverse environmental and physiological conditions.

While the present approach focuses on ECG, EEG, and respiratory signals, the underlying principles of the proposed approach are designed to be versatile and adaptable to a broad spectrum of biosignals. The methodology relies on fundamental signal processing that is applicable across different physiological data types. However, empirical validation on a wider range of biosignals such as transcutaneous CO2, SpO2, (non)invasive blood pressure, intracranial pressure, and galvanic skin responses, is required.

Furthermore, we combined several unimodal SQIs for deciding if a certain segment is suitable for multimodal analysis. It would be more advantageous to use SQIs that are adopted for multimodal signals but the authors are not aware that these exist. In addition, physiological SQIs were applied exclusively to ECGs as they do not exist for EEG or respiration signals. Hence, a potential avenue for future work would be to develop multimodal SQIs for a more accurate analysis of suitability. However, due to the generalizability of the used methods, the proposed analysis and its results can probably be generalized to any biosignal dataset other than the considered VitalDB and SIESTA datasets with only the physiological SQIs being restricted to ECG signals.

### E. Scope and Future Work

The results of our study are important from two main perspectives. On one hand, it provides valuable information to data scientists and machine learning experts on how much data of VitalDB is suitable for multimodal analysis. In addition, the proposed methodology offers a straightforward approach to detecting unsuitable signal segments that can be excluded for further analysis. For instance, this is an important step in neural networks as they are susceptible to noisy labels, outliers, and corrupted data during training, which can compromise generalization and robustness [65]. On the other hand, our histogram analysis revealed a correspondence between quantitative statistical SQIs (i.e. entropy outliers) and qualitative physiological SQIs, which warrants further research in this direction. Additionally, we will implement data-driven feature ranking of the SQIs in future work, which will allow us to understand how each feature contributes to the description of signal suitability.

## V. Conclusion

We analyzed the suitability of VitalDB, a dataset consisting of biosignals recorded during surgery for multimodal signal analysis. The results indicate that its suitability is 10% reduced in comparison to SIESTA, a sleep dataset. Sleep datasets have already been used successfully for different multimodal analysis [3], [5], [6], [7], which underlines the potential of using VitalDB for similar analyses. Most of the SQIs utilized in this study are not signal- or measurement-specific and therefore the proposed methodology could be applied to any biosignal dataset by other researchers to compare its suitability, using the values reported in this paper for sleep and surgery datasets as a reference.

## Author contributions

**Ennio Idrobo-Ávila:** Conceptualization, methodology, software, formal analysis, validation, investigation, writing original draft, and writing - review & editing. **Gergő Bognár:** Methodology, software, investigation, writing original draft, and writing - review & editing. **Dagmar Krefting:** Conceptualization, validation, and funding acquisition. **Thomas Penzel:** Validation and manuscript reviewing. **Péter Kovács:** Methodology, validation, investigation, writing original draft, and writing - review & editing. **Nicolai Spicher:** Conceptualization, methodology, validation, investigation, writing original draft, writing - review & editing, and project administration.

## Conflict of interest

All authors declare no conflicts of interest in this manuscript.
